# Utilizing Fly Ash from Coal-Fired Power Plants to Join ZrO_2_ and Crofer by Reactive Air Brazing

**DOI:** 10.3390/ma18091956

**Published:** 2025-04-25

**Authors:** Shu-Wei Chang, Ren-Kae Shiue, Liang-Wei Huang

**Affiliations:** 1 Department of Materials Science and Engineering, National Taiwan University, Taipei 106, Taiwan; d03527009@ntu.edu.tw; 2 Chemistry and Environment Research Laboratory, Taiwan Power Research Institute, Taiwan Power Company, New Taipei City 238, Taiwan; 3 Department of Material Research, National Atomic Research Institute, Taoyuan 325, Taiwan; i13501350@nari.org.tw

**Keywords:** fly ash, reactive air brazing, airtightness, microstructure, interface

## Abstract

This study attempts to use fly ash as the brazing filler additive to increase the sustainable use of coal-fired power plant by-product materials. The experimental results show that adding 5 wt% fly ash into the Ag paste filler contributes to the interfacial reactions in heterogeneous reactive air brazing (RAB) of the ZrO_2_ and Crofer alloy. The Ag-rich phase dominates the brazed zone. The interfacial reaction layers contain oxidation of the Cu-Ti coating layer, Crofer alloy, and the Si/Al-rich oxides from the fly ash particles. The 5% fly ash RAB joint maintained airtightness for 280 h under 2 psig helium at room temperature. When the test temperature was raised to 600 °C for 24 h, the pressure of the joint assembly still did not drop. When the fly ash addition was increased to 10 wt%, the joint assembly was no longer leak-free at room temperature. Many visible voids and cracks exist in the brazed zone and at the ZrO_2_/braze and braze/Crofer interfaces. A high volume fraction of the fly ash particles results in many brittle Si/Al-rich oxides in the joint after RAB, and the fracture of these oxides significantly deteriorates the airtightness of the joint. This study shows the feasibility and potential of introducing 5 wt% fly ash particles to the Ag-rich paste filler during the RAB of ZrO_2_ and Crofer for airtight applications.

## 1. Introduction

Regarding power generation technology, coal-fired power generation is one of the world’s primary methods. Even with the trend of energy conservation and carbon reduction, the International Energy Agency (IEA) still predicts that the global demand for coal will continue to grow before 2027 [1]. To improve combustion efficiency, coal-fired power plants grind coal into powder before it is sent to the boiler for combustion. The solid residue after combustion is called coal ash, and the heavier particles that fall to the bottom of the furnace, called bottom ash, account for about 5 to 20 wt% of the total fly ash. The lighter particles carry the flue gas to the electrostatic precipitator or bag filter. The dust particles collected by dust collectors or bag filters are called fly ash, which accounts for about 80–95 wt% of the total coal ash [2,3].

The physical and chemical properties of coal ash often vary depending on factors such as the power plant unit, coal source used, pulverized coal fineness, and boiler combustion conditions [4,5]. Fly ash is the solid residue left after coal combustion. After the coal mineral material is burned, the fly ash contains a lot of SiO_2_, Al_2_O_3_, and Fe_2_O_3_ [6,7]. The main characteristic of coal is spherical particles, caused by the high-temperature combustion of coal powder at about 1400 to 1700 °C [8,9]. Fly ash can be considered power plant waste, but many examples show fly ash being reused. Some countries’ fly ash utilization rates can reach 100% [2]. The main reason for this is that fly ash undergoes a pozzolanic reaction that can replace part of the cement or fill fine aggregate. When added to concrete appropriately, it can improve workability, reduce hydration heat, increase late strength, enhance durability, and strengthen sulfate resistance [10,11]. In the past, applications and research were mostly related to concrete and cement, mainly used in the production of building materials, paving [12], production of cement products [13], soil improvement, land reclamation, and road construction [14].

Previous studies have shown that the main components of power plant fly ash are SiO_2_ and Al_2_O_3_, of which SiO_2_ and Al_2_O_3_ together account for about 80% [15]. Therefore, in terms of material properties, it can be regarded as a ceramic material. Fly ash has a certain degree of fineness and is mainly spherical in appearance, which makes it suitable for use as an additive in materials processing. Although the engineering application of fly ash is widespread, similar applications have not yet been reported in the dissimilar joining process. Adding fly ash as filler in the material manufacturing process shows research and development potential.

Solid oxide fuel cells (SOFCs) have many advantages in power generation. First, solid oxide fuel cells have the benefits of high energy efficiency and the ability to use a variety of fuels. In addition, they can be used as an independently operated, distributed power generation system to reduce the energy loss of the central power supply system during power transportation [16,17,18]. The body of the SOFC is made of ceramics, and the collector plate is mainly made of metallic alloy. The sealing technology between metal and ceramics becomes the key issue as to whether the SOFC can operate safely and stably [19,20,21]. The three most commonly used sealing materials are glass, ceramic, and pressure-resistant metal [22]. Many SOFC sealing approaches have been studied, including rigid glass, glass/ceramic, fiber-reinforced glass seals, compressed mica seals, and metal seals such as active brazing [22]. Most non-metallic sealing materials have poor thermal stability in high-temperature environments, causing the structure to swell. This can lead to structural degradation and airtight failure of the SOFC, and its thermal cycling stability is also poor [23,24].

Compared with other sealing technologies, dissimilar brazing technology can provide relatively good airtightness, conductivity, and better long-term thermal stability during the operation of SOFC. It is one of the sealing processes with development potential at present [25,26,27]. The most commonly known brazing techniques for SOFC sealing are active metal brazing (AMB) and reactive air brazing (RAB). A significant concern in AMB is the need to use an inert atmosphere (e.g., Ar, He) and the relatively high vacuum (<10^−5^ mbar) required during the sealing process. The low-oxygen partial pressure required during active brazing may lead to irreversible degradation of the ceramics [28]. In recent years, some scholars have tried to improve the wettability of the material surface by metalizing ceramics to improve the sealing performance of the brazing process [29]. Scholars have also tried using commercial active braze alloys with low melting points to join metal and ceramics [30,31]. Silver has been proposed as the basic sealing material by introducing some oxides to improve the thermal expansion mismatch in the dissimilar joint [32,33,34]. This way, ZrO_2_ and Crofer alloy were successfully joined with good airtightness at high temperatures [35].

The brazing filler can also use Ag to introduce fly ash, a byproduct after high-temperature coal combustion at the coal power plant. This study will try to use fly ash as a filler additive to perform the dissimilar RAB of ZrO_2_ and Crofer alloy. The feasibility of its application in high-temperature airtightness is also evaluated. In addition to improving material costs, it helps recycle and sustainably utilize power plant by-products, such as fly ash.

## 2. Materials and Experimental Procedures

### 2.1. Materials

ZrO_2_ (Kceracell, Geumsan-gun, Republic of Korea) and Crofer alloy (VDM Metals, Werdohl, Germany) were substrates in the experiment. The Ag-based brazing alloy used in this study was a paste-type filler containing Ag powder. Its name is PELCO^®^ colloidal silver. The Ag base was 60 ± 1 wt%. The paste included toluene, ethyl acetate, acetate, ethanol, n-butyl acetate, isopropyl alcohol, propan-2-ol, trimethyl bicyclo, and other organic substances. Fly ash was used as the filler additive to further reduce material costs and highlight the spirit of sustainability. The fly ash was taken from the Taiwan Fossil Power Plant in Linkou City, Taiwan. Two proportions of fly ash (5 and 10 wt%) were pre-added to the PELCO^®^ colloidal silver.

**Figure 1:**
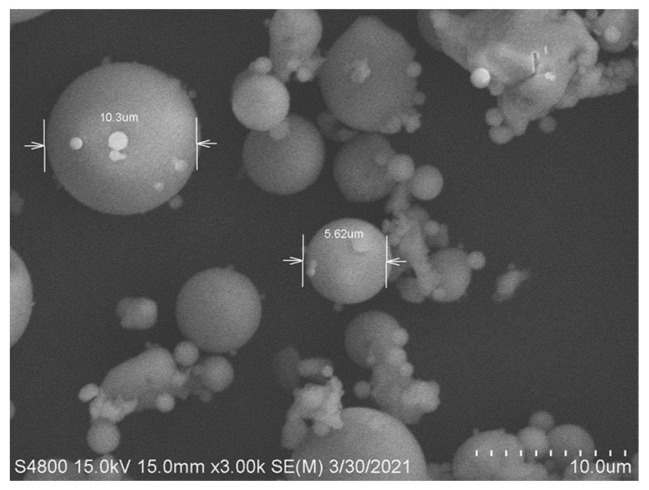
The morphology of fly ash used in the experiment.

**Figure 2:**
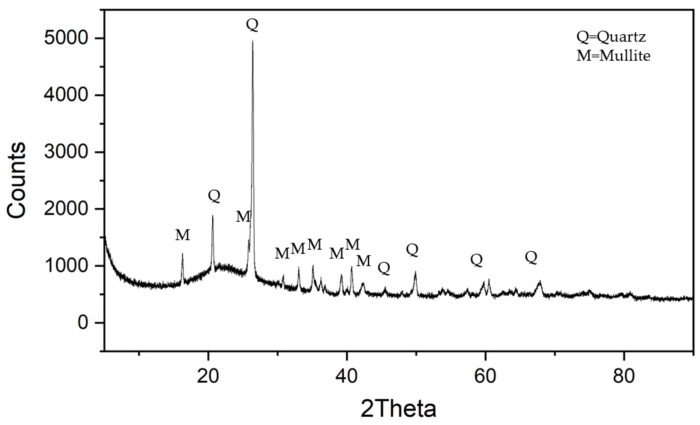
Fly ash XRD analysis results.

The microscopic appearance of the fly ash after 200-mesh screening is shown in Figure 1. The fly ash is spherical, ranging between 1 and 10 µm in diameter. The exact chemical composition of fly ash depends primarily on the coal burned. In general, the chemical composition of fly ash is composed of elements such as C, O, Na, Al, Si, Fe, and Ca, with silicon oxide and aluminum oxide as the main components [6,7,8]. The results of the inductively coupled plasma (ICP) chemical element analysis are shown in Table 1. It is worth mentioning that the sum of all contents is 104.6, higher than 100, because all metals are considered as oxides in Table 1. The dry-basis fly ash mainly comprises SiO_2_ and Al_2_O_3_. Figure 2 displays the XRD spectrum of the fly ash. The main crystal structures in fly ash are quartz (SiO_2_) and mullite (3Al_2_O_3_·2SiO_2_). The result is similar to reports in the literature [36].

### 2.2. Sputtering of Two Substrates

**Figure 3:**
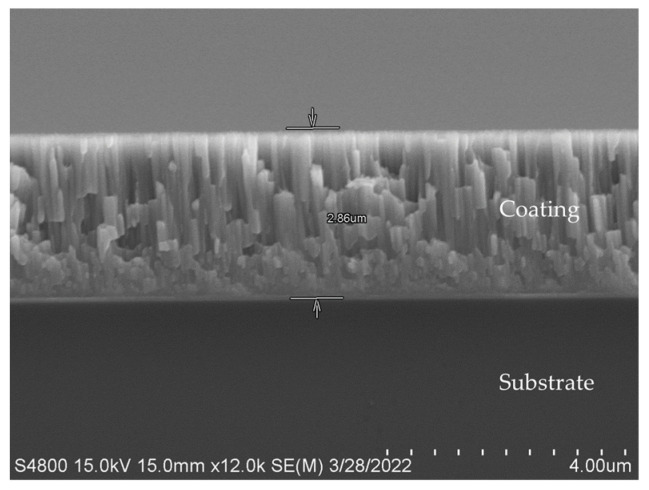
The coating thickness of the 70Cu-30Ti alloy after sputtering.

ZrO_2_ and Crofer alloy were metalized using a sputtering machine (KaoDuen KD-SPA01, New Taipei City, Taiwan). Both substrates were preheated to 200 °C under a vacuum of 6 × 10^−6^ mbar, and Ar was introduced to a pressure of 3 × 10^−2^ mbar. The rotating disk temperature was controlled at 200 °C, and the powers of dual targets were 170 W (Ti) and 80 W (Cu), respectively. The thickness of the coating layer (~3 μm) was controlled by sputtering time (2 h). The film thickness was measured by an electron microscope (Hitachi S-4800, Tokyo, Japan), and an EDS (Energy Dispersive Spectrometer, Bruker-Quantax, Berlin, Germany) was used to perform the cross-section’s microscopic and chemical composition analysis. The coating thickness after sputtering is shown in Figure 3. The chemical composition of the central coating layer is 70.2 Cu and 29.8 Ti in at%. According to the Cu-Ti binary alloy phase diagram, the solidus and liquidus temperatures of the coated layer are 870 and 900 °C [37].

### 2.3. Sample Preparation and Analyses After Brazing

In preparing the brazing sample, the ZrO_2_ and Crofer alloy were first ground with silicon carbide sandpaper of 1000 grit to remove surface contamination. Then, the test piece was immersed in an alcohol solvent. The contamination on the surface of the specimen was removed by ultrasonic vibration and finally dried for use. The filler paste on Crofer metal’s and YSZ ceramic’s surface was fixed with alumina fixtures, assembled into a sandwich structure, and then heated to 960 °C at 60 °C/h in the air. After maintaining the temperature for 1200 s, the temperature was lowered to room temperature at 60 °C/h. The brazed test piece was cut slowly with a diamond saw to obtain its cross-section, then hot-embedded with the conductive epoxy resin, and then ground and polished. The alumina polishing particles were 9 μm, 3 μm, and 1 μm, respectively. Then, the cross-section microstructure and chemical composition analysis were performed using an electron microscope (Hitachi S-4800, Tokyo, Japan) and an energy dispersive spectrometer (Bruker-Quantax, Berlin, Germany). Quantitative chemical analyses and mappings were performed using an electron probe microanalyzer (EPMA), JEOL JXA-8530F Plus (Tokyo, Japan).

**Figure 4:**
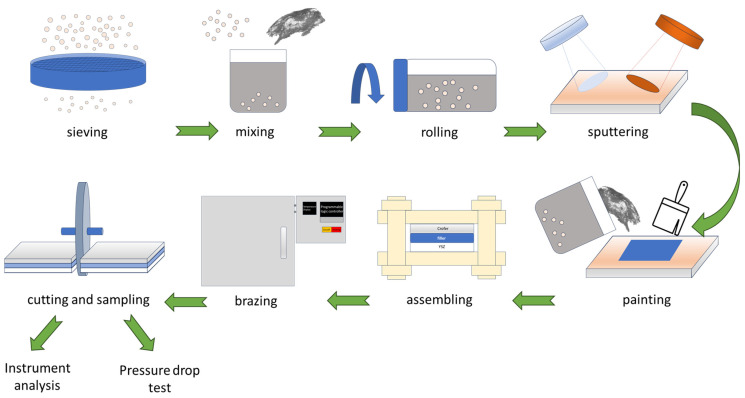
The schematic diagram of the experimental procedure.

In summary, the fly ash was sieved below 200 mesh and mixed with Ag paste to form a Ag-based brazing paste. A rolling mixer was applied to the Ag-based paste to make the fly ash uniformly distributed in the paste. ZrO_2_ and Crofer substrates were coated with a layer of 70.2% Cu-29.8% Ti, approximately 3 μm thick. The Ag-based paste was painted on the ZrO_2_ substrate. The painted ZrO_2_ and Crofer alloy were fixed using an alumina fixture and put in a box furnace. The reactive air brazing was performed in the furnace. After brazing, the brazed joint was cut using a slow-speed diamond saw. The reactive brazed joint was prepared for the instrument analysis and pressure drop test. A schematic diagram of the experimental procedure is shown in Figure 4.

### 2.4. Airtight Test

The pressure drop method was used to measure the pressure change in the sample to evaluate the airtightness of the RAB joint. The ZrO_2_ and Crofer plates had sizes of 20 × 20 × 2 mm^3^ and 14 × 14 × 2 mm^3^, respectively. A hole with a diameter of 8 mm was drilled in the center area of the Crofer plate in advance and connected to the gas source through a metal tube. After the sample was brazed, the 310 stainless steel tube was laser welded to the drilled hole. The tube was connected to the helium source. The internal pressure of the assembly was recorded over time, and the leakage rate of the brazed joint was obtained.

## 3. Results and Discussion

### 3.1. Microstructural Observation of the RAB Joint with 5 wt% Fly Ash Addition

**Figure 5:**
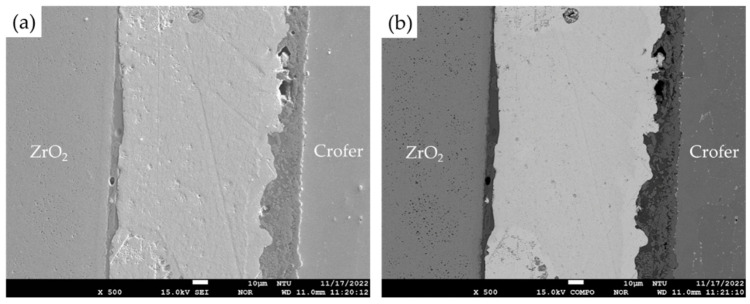
EPMA (**a**) SEI, (**b**) BEI of the brazed zone with the 5% fly ash addition.

The EPMA observations and chemical analysis of the 5 wt% fly ash RAB joint were performed in the experiment. The SEI (secondary electron image) and BEI (backscattered electron image) of the brazed zone are shown in Figure 5. The spherical fly ash is not observed in the brazed zone after brazing in the air. There was no separation of spherical fly ash in the brazed zone. Additionally, no interfacial separation was found after brazing. The interfaces of the ZrO_2_ side and Crofer side contain a few voids, but there is no long crack along both interfaces. This demonstrates the potential for the subsequent airtightness test.

**Figure 6:**
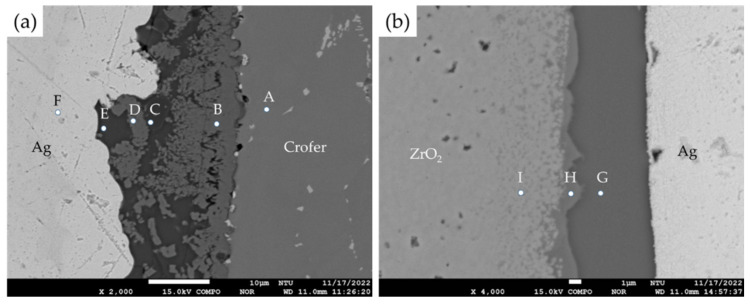
The EPMA BEIs and quantitative chemical analyses of various phases in the brazed zone: (**a**) the Crofer side, (**b**) the ZrO_2_ side.

Figure 6 shows the EPMA quantitative chemical analyses in at% during various phases after brazing in the Crofer and ZrO_2_ sides, respectively. The oxidation and interfacial reactions between the fly ash and 70Cu-30Ti coated layer on both sides are apparent. For the Crofer side, the interface’s thickness is approximately between 10 and 20 µm, as displayed in Figure 6a. The interface near the Crofer side is mainly composed of Al/Cr/Cu/Si/Ti-rich oxides, as marked by B~E in Figure 6a. The center of the brazed zone in Figure 6a is the Ag-rich braze, as marked by F. There is a Si/Al-rich oxide, as marked by E at the interface close to the Ag-rich braze. Table 1 and Figure 2 show that the fly ash mainly consists of quartz (SiO_2_) and mullite (3Al_2_O_3_·2SiO_2_). The chemical composition of Si/Al-rich oxides is close to that of fly ash. It is deduced that the fly ash reacts with the oxidized 70Cu-30Ti coated layer during the air brazing, and a sound joint between the Ag-rich braze and Crofer substrate is achieved.

**Figure 7:**
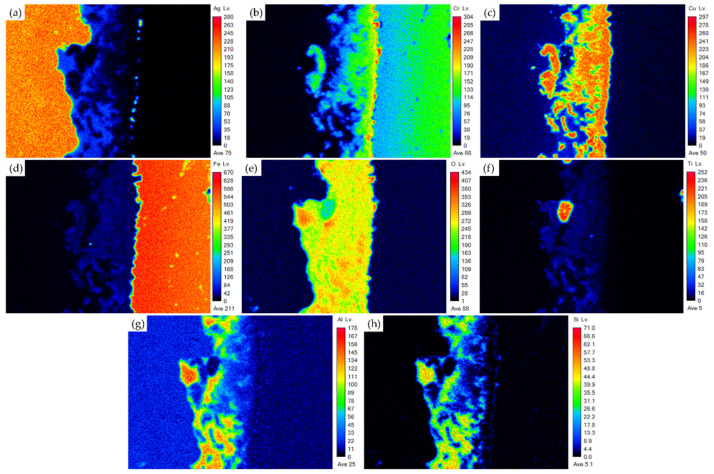
The EPMA element mappings of the Crofer side in Figure 6a: (**a**) Ag, (**b**) Cr, (**c**) Cu, (**d**) Fe, (**e**) O, (**f**) Ti, (**g**) Al, (**h**) Si.

Figure 7 shows the EPMA element mappings of the Crofer side in Figure 6a. The interfacial reaction layer contains a high content of oxygen (Figure 7e) and comprises oxides. The distributions of Cr and Cu overlap, as displayed in Figure 7b,c. The Cr/Cu-rich oxides are consistent with positions B and D of the quantitative chemical analysis results shown in Figure 6a and Table 2. It is deduced that oxidations of the 70Cu-30Ti coating layer and the Cr in the Crofer substrate result in the formation of Cr/Cu-rich oxides. It is also noted that the dissolution of Crofer into the molten braze and Cr oxidation are observed in Figure 7b during air brazing. Additionally, the distributions of Al and Si are almost identical, as displayed in Figure 7g,h. The Si/Al-rich oxides originate from the fly ash. After air brazing, the interfacial reaction layer comprises the fly ash, the oxidized 70Cu-30Ti coating layer, and the Cr oxide in the Crofer substrate. Introducing the fly ash into the Ag paste works well in air brazing the Crofer alloy.

The microstructure of the ZrO_2_ side is well bonded, as displayed in Figure 6b. The thickness of the interface between the braze alloy and ZrO_2_ substrate is between 5 and 10 µm. In Figure 6b, a thin Cu/Fe/Ti/Si-rich oxide layer, as marked by H, readily wets and bonds to the ZrO_2_ substrate. The 70Cu-30Ti coated layer present during air brazing was oxidized, and the ZrO_2_ surface was wet well. The chemical composition of the darker area of the interfacial layer is mainly composed of Si/Al-rich oxides, as marked by G in Figure 6b. The fly ash primarily comprises SiO_2_ and Al_2_O_3_. It is melted, forming a continuous interfacial layer between the Ag-rich braze and ZrO_2_ substrate.

**Figure 8:**
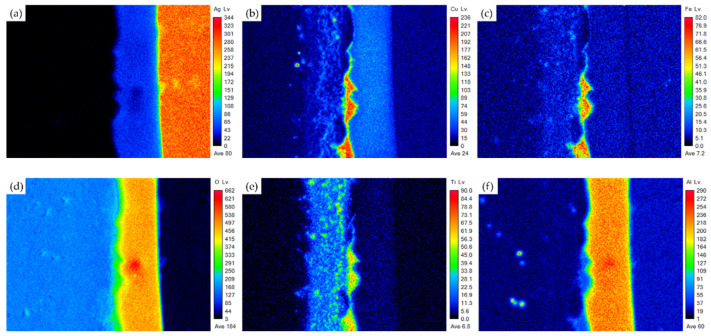

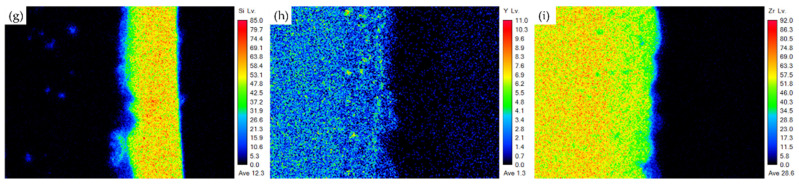
The EPMA element mappings of the ZrO_2_ side in Figure 6b: (**a**) Ag, (**b**) Cu, (**c**) Fe, (**d**) O, (**e**) Ti, (**f**) Al, (**g**) Si, (**h**) Y, (**i**) Zr.

Figure 8 shows the EPMA element mappings of the ZrO_2_ side in Figure 6b. In Figure 8d, the interfacial reaction layer has a high oxygen concentration. The distribution of Cu, Fe, and Ti in element mappings overlaps, as shown in Figure 8b,c,e. Therefore, they are Cu/Fe/Ti-rich oxides. The content of these oxides is consistent with the quantitative chemical analysis at position H, as shown in Figure 6b and Table 2. It is noted that the fly ash contains 6.69 wt% Fe_2_O_3_ (Table 1), and it contributes to the wetting of the ZrO_2_ surface in air brazing. The reaction between the fly ash and oxidized 70Cu-30Ti coating layer results in thin Cu/Fe/Ti/Si-rich oxides on the surface of ZrO_2_. It provides a wettable surface on the ZrO_2_. In Figure 8d,f,g, the mappings of Al and Si coincide with that of O, and the Si/Al-rich oxides originate from the fly ash, as illustrated by Figure 6b and Table 2. After brazing, the fly ash forms a layer on the wettable surface of Cu/Fe/Ti/Si-rich oxides. Adding 5 wt% fly ash into the Ag braze promotes the wetting of the ZrO_2_ substrate in air brazing. Introducing 5 wt% fly ash into the Ag paste benefits the dissimilar reactive air brazing of the ZrO_2_ and Crofer. Reactive wetting on both the Crofer and ZrO_2_ sides was successfully achieved.

The addition of 5 wt% fly ash into the Ag paste significantly affects the microstructures of the Crofer/braze and ZrO_2_/braze interfaces. Although there are a few voids at the interfaces, the interfaces are well bonded, without long cracks along these interfaces. Additionally, there is no spherical fly ash particle alone in the Ag-rich braze. The main ingredients of the fly ash are quartz (SiO_2_) and mullite (3Al_2_O_3_·2SiO_2_). The thermal coefficient of the fly ash is quite different from that of the Ag-rich filler. The presence of unreacted fly ash in the Ag-rich braze is detrimental to the air-tight performance of the joint.

### 3.2. Microstructural Observation of the RAB Joint with 10 wt% Fly Ash Addition

**Figure 9:**
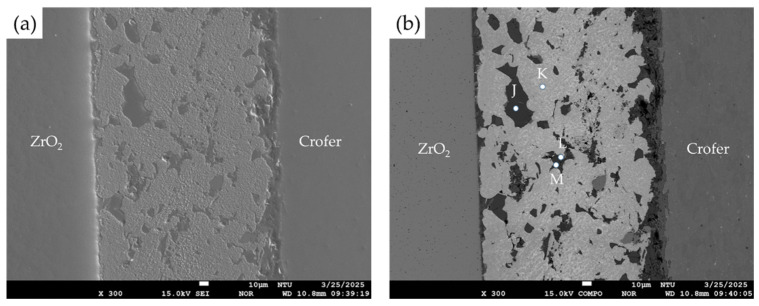
EPMA (**a**) SEI, (**b**) BEI of the brazed zone with the 10% fly ash addition.

**Figure 10:**
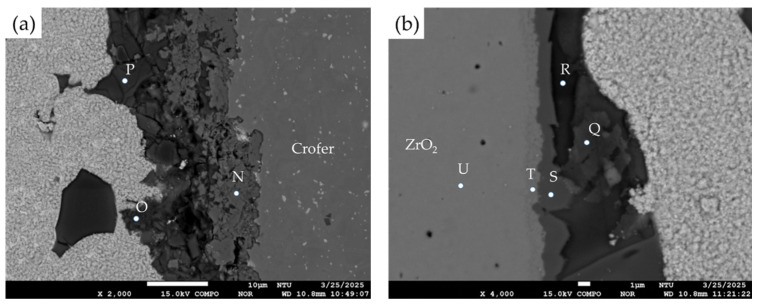
The EPMA BEIs and quantitative chemical analyses of various phases in the brazed zone: (**a**) the Crofer side, (**b**) the ZrO_2_ side.

Figure 9 shows the SEI and BEI of the RAB joint with the 10% fly ash addition. The microstructure of Figure 9 is very different from that of Figure 6. Although introducing 10 wt% fly ash into the Ag paste could successfully result in the RAB of ZrO_2_ and Crofer, many visible voids were observed in the brazed zone. Additionally, many unreacted fly ash particles remained in the brazed zone. Figure 10 shows the EPMA BEIs and quantitative chemical analyses of various phases on the Crofer and ZrO_2_ sides, respectively. The EPMA quantitative chemical analyses of the multiple phases in Figure 9 and Figure 10 are displayed in Table 3. In Figure 9b, the brazed zone contains Ag-rich braze (location K), Si/Al-rich oxides (location J and M), and Cu-rich oxide (location L). It is noted that 10 wt% fly ash addition to the Ag-rich paste results in many irregular-shaped fly ash particles in the brazed zone after RAB. The irregular-shaped fly ash particles are brittle, and many voids result from the fracture of these fly ash particles.

In Figure 10a, quantitative chemical analyses of various phases at the interface of the Crofer side were completed. The Cr/Cu-rich oxides (location N) are next to the Crofer substrate. The oxidation of the Cu-Ti coated layer and Crofer substrate is apparent. A layer of Si/Al-rich oxides (location O and P) is deposited on the interfacial Cr/Cu-rich oxides. It is worth mentioning that there are many cracks and cavities in the Si/Al-rich oxides due to the inherent brittleness of the fly ash. Figure 10b shows quantitative chemical analyses of various phases of the ZrO_2_ side. There is a layer of Cu/Fe/Ti/Si-rich oxides (location S) on the surface of the ZrO_2_ substrate (location T and U), and it provides a wettable surface to the fly ash (location Q and R).

### 3.3. Airtightness Test of the Air-Brazed Joint

**Figure 11:**
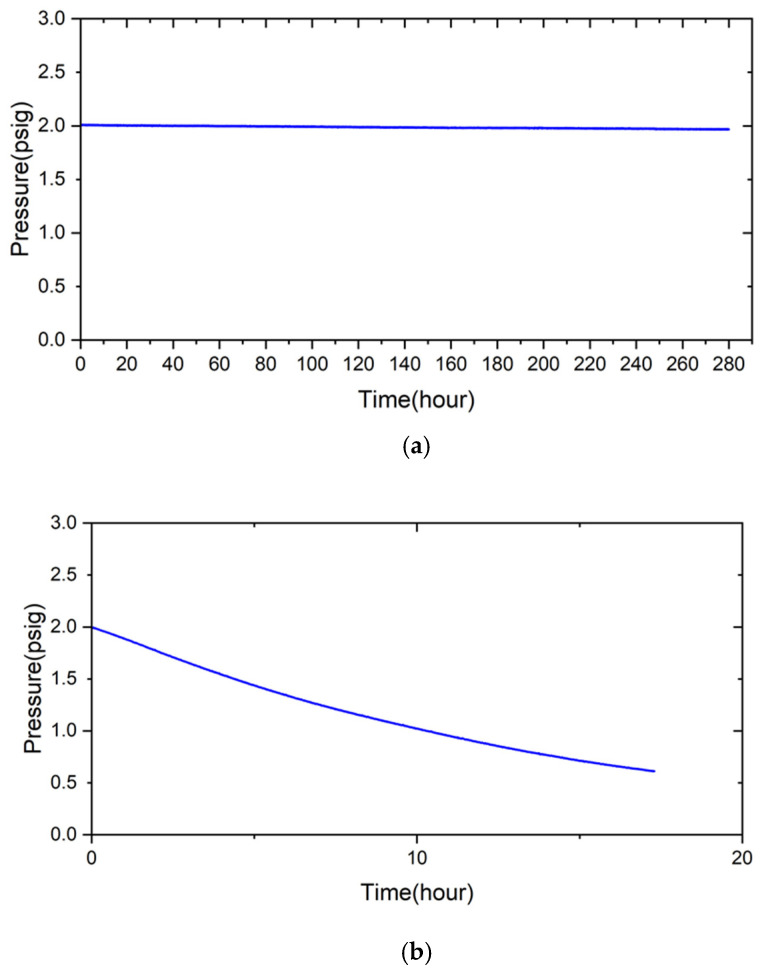
The pressure drop test result of the RAB joint with (**a**) 5 wt%, (**b**) 10 wt% fly ash additions at room temperature.

Figure 11 shows the pressure drop test results of the RAB joints with 5 and 10 wt% fly ash additions at room temperature. In Figure 11a, the condition of 2 psig helium at room temperature was maintained for a total of 280 h, without any decrease in pressure for the 5 wt% fly ash addition brazed joint. In contrast, the airtightness of the RAB joint with 10 wt% fly ash addition showed very poor performance. The pressure was decreased from 2 psig at the beginning of the pressure drop test, and the gauge pressure was 1 psig after the 10 h test. Since the fly ash was brittle, a high volume fraction of fly ash addition into the Ag-rich filler paste resulted in the fracture of the Si/Al-rich oxides, the primary ingredients of the fly ash, after RAB.

**Figure 12:**
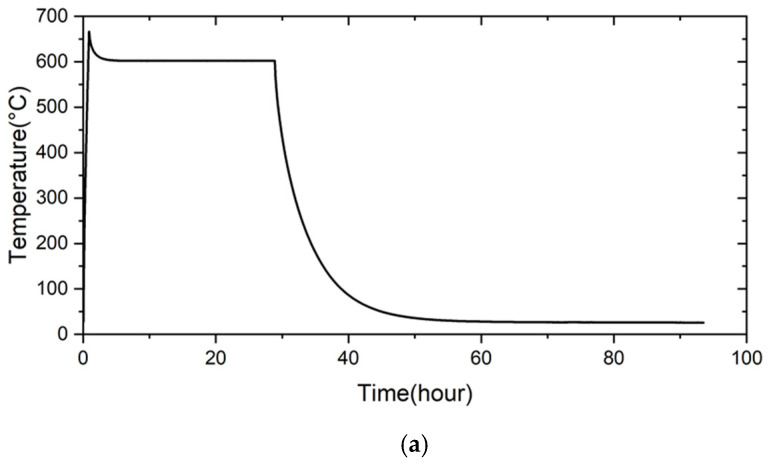

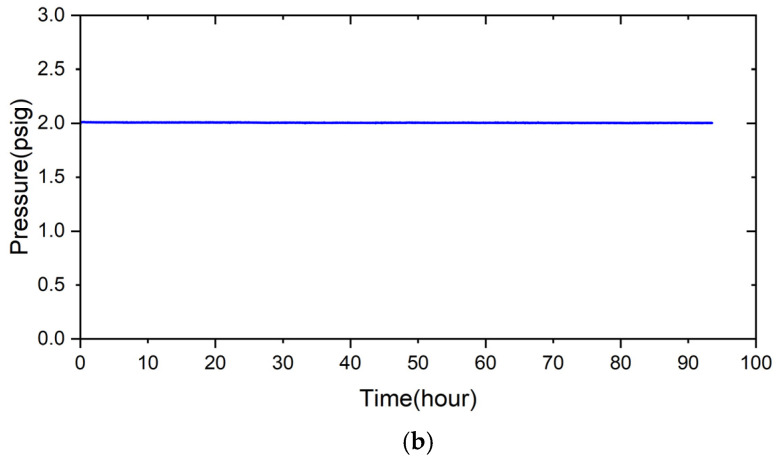
The pressure drop test result of the RAB joint with 5 wt% fly ash addition at 600 °C: (**a**) temperature contour, (**b**) pressure contour.

The pressure drop test of the RAB joint with 5 wt% fly ash was further tested at elevated temperatures. Figure 12 shows the pressure drop result of the RAB joint with 5 wt% fly ash addition at 600 °C. The initial pressure of helium before the test was 2.01 psig. The pressure was not changed after heating to 600 °C and holding for 24 h. The pressure was maintained upon subsequent cooling to room temperature for 95 h. The brazed joint showed good airtightness for the complete thermal cycle of the test. Compared to prior studies, the current experimental result shows the feasibility and potential of introducing fly ash into Ag-rich paste filler to perform the RAB of ZrO_2_ and Crofer alloy in future gas-tight applications [31,35].

**Figure 13:**
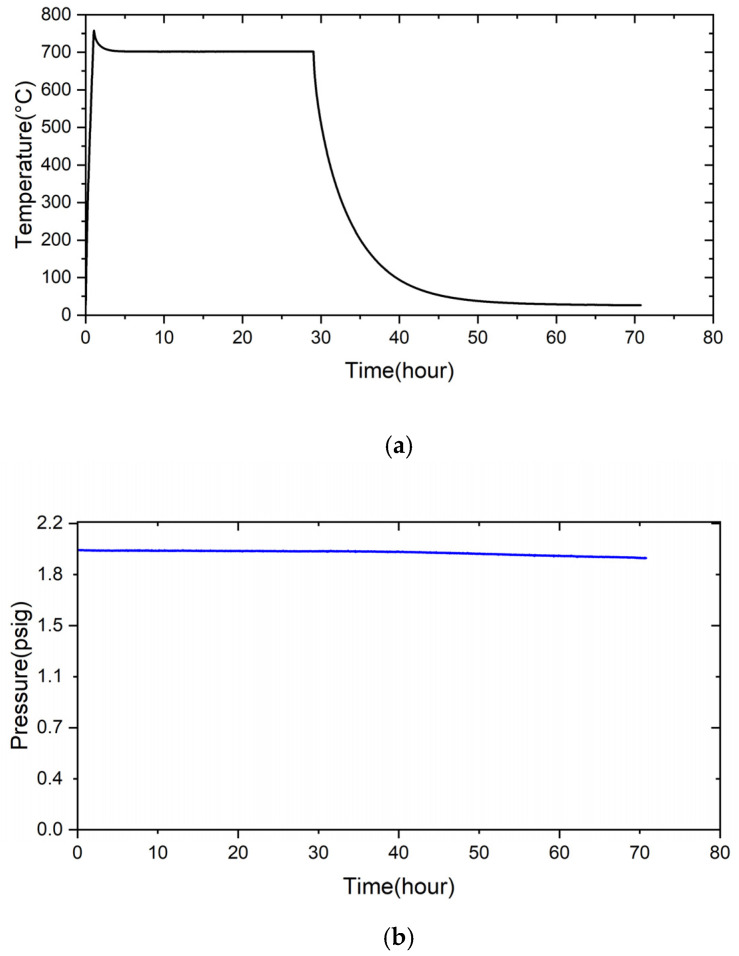
The pressure drop test of the air-brazed joint with 5 wt% fly ash addition at 700 °C: (**a**) temperature contour, (**b**) pressure contour.

Figure 13 shows the pressure drop test result of the RAB joint with 5 wt% fly ash addition at 700 °C. The initial pressure was 2.00 psig. After heating to 700 °C and holding the temperature for 24 h, the pressure value dropped slightly to 1.943 psig. The average slope of the pressure drop was 8.14 × 10^−4^ psig/h. The airtightness of the joint deteriorated at 700 °C.

Reactive air brazing at 960 °C for 1200 s to join Cu-Ti metalized ZrO_2_ and Crofer alloy using the Ag paste without fly ash was performed in a previous study [35]. The optimized Cu-Ti coating layer was 3 μm in thickness. The Cu-Ti coated layer on the ZrO_2_ substrate resulted in a defect-free interface between the Ag-rich braze and the ZrO_2_ [35]. The pressure drop test displayed no leakage under 2 psig at room temperature for 28 h or at 600 °C for 24 h, respectively [35]. Additionally, the reactive brazed joint that used Ag paste with 5 wt% fly ash demonstrated comparable performance in the airtightness test. Adding fly ash into the Ag-based paste presents the advantages of promoting sustainability and recycling waste material.

According to the experimental results, adding 5 wt% fly ash into the Ag-based paste for the reactive air brazing of 70Cu-30Ti metallized ZrO_2_ can promote surface wetting of the ZrO_2_ substrate, and an airtight joint can be achieved. It is deduced that a similar approach can be applied to the reactive air brazing of many other ceramics, e.g., Al_2_O_3_, MgO, Si_3_N_4_. It presents a potential method to increase the sustainable use of coal-fired power plant by-product materials, such as fly ash.

## 4. Conclusions

This study used fly ash as a Ag-rich filler paste additive to perform heterogeneous RAB of ZrO_2_ and Crofer alloy. The study shows the feasibility and potential of introducing fly ash particles to Ag-rich paste filler to braze ZrO_2_ and Crofer for airtight applications. The microstructure and airtightness of the joint were evaluated in the experiment. The important conclusions are shown below.

1. The Ag-rich phase dominates the brazed zone in the 5% fly ash RAB joint. The interfacial reaction layers contain the oxidation of the Cu-Ti coating layer, Crofer alloy, and the Si/Al-rich oxides from the fly ash particles. Good bonding among the ZrO_2_, Ag-rich braze with 5 wt% fly ash and Crofer alloy was achieved in the experiment.
2. The 5% fly ash RAB joint maintained airtightness for 280 h under 2 psig helium at room temperature. When the test temperature was raised to 600 °C for 24 h, the pressure of the joint assembly still did not drop. After the test was performed at 700 °C for 24 h, the pressure of the joint assembly decreased slightly, with a pressure drop slope of 8.14 × 10^−4^ psig/h.
3. When the fly ash addition was increased to 10 wt%, the joint assembly was no longer leak-free at room temperature. There were many visible voids and cracks in the brazed zone and at both interfaces, ZrO_2_/braze and braze/Crofer. A high volume fraction of the fly ash particles results in many brittle Si/Al-rich oxides in the joint after RAB, and the fracture of these oxides significantly deteriorates the airtightness of the joint.
4. Compared with the reactive air brazed joint using Ag paste without fly ash, the brazed joint with 5 wt% fly ash Ag-rich filler paste demonstrated comparable performance in the airtightness test. Furthermore, adding fly ash into the Ag-based paste presents the advantages of promoting sustainability and recycling waste materials.

## Figures and Tables

**Table 1:** ICP analysis result of the fly ash.

Moisture content (%)		0.11
Burn reduction (%)		2.75
Composition on dry basis (%)	Al_2_O_3_	22.5
	CaO	4.44
	CuO	<0.02
	Fe_2_O_3_	6.69
	K_2_O	1.12
	MgO	1.72
	Na_2_O	1.10
	SiO_2_	64.1
	TiO_2_	0.94
	V_2_O_5_	<0.03
Unburned Carbon (%)		1.96

**Table 2:** EPMA WDS quantitative chemical analyses in at% of the reactive air-brazed joint in Figure 6.

Element	Ag	Cu	Cr	Fe	O	Ti	Y	Zr	Si	Al	Alloy/Phase
A	0.0	0.0	23.2	73.8	0.0	0.0	0.0	0.0	0.0	0.0	Crofer substrate
B	0.3	24.9	21.6	1.9	48.7	0.9	0.0	0.0	0.4	0.6	Cr/Cu-rich oxides
C	1.6	0.8	0.4	0.3	63.7	19.6	0.0	0.1	9.6	2.7	Ti/Si/Al-rich oxides
D	0.2	26.0	20.5	1.3	48.8	1.7	0.0	0.0	0.1	0.5	Cr/Cu-rich oxides
E	2.5	0.7	0.0	0.1	66.7	0.2	0.0	0.0	22.9	6.2	Si/Al-rich oxides
F	99.6	0.0	0.0	0.0	0.1	0.1	0.0	0.0	0.0	0.0	Ag-rich braze
G	2.5	2.4	0.0	0.2	63.1	0.3	0.0	0.0	23.3	7.8	Si/Al-rich oxides
H	0.0	14.9	0.7	4.1	56.7	7.9	1.3	4.6	5.7	2.7	Cu/Fe/Ti/Si-rich oxides
I	0.0	0.9	0.0	0.3	62.2	2.9	1.3	31.9	0.0	0.1	ZrO_2_ alloyed with Ti

**Table 3:** EPMA WDS chemical analyses in at% of the reactive air-brazed joint in Figure 9 and Figure 10.

Element/at%	Ag	Cu	Cr	Fe	O	Ti	Y	Zr	Si	Al	Alloy/Phase
J	3.5	3.5	0.0	0.5	61.8	0.7	0.0	0.0	21.9	7.8	Si/Al-rich oxides
K	97.4	0.0	0.0	1.9	0.4	0.0	0.0	0.0	0.0	0.0	Ag-rich braze
L	0.1	55.0	0.0	0.0	44.6	0.0	0.0	0.0	0.0	0.0	Cu-rich oxide
M	6.2	1.2	0.0	0.6	62.8	1.1	0.0	0.0	20.6	7.1	Si/Al-rich oxides
N	3.6	18.2	11.2	2.4	48.7	1.8	0.0	0.0	8.7	4.5	Cr/Cu-rich oxides
O	5.9	3.9	0.5	8.9	53.9	0.5	0.0	0.0	14.5	11.3	Si/Al-rich oxides
P	5.9	1.1	0.0	0.3	62.2	0.3	0.0	0.0	22.0	7.8	Si/Al-rich oxides
Q	6.5	2.6	0.7	0.6	53.4	0.5	0.0	0.0	26.3	9.7	Si/Al-rich oxides
R	4.8	1.4	0.0	0.5	57.7	0.5	0.0	0.0	25.0	9.5	Si/Al-rich oxides
S	0.0	17.9	0.3	5.8	51.9	9.2	0.9	4.4	5.2	3.8	Cu/Fe/Ti/Si-rich oxides
T	0.0	0.8	0.0	0.4	58.4	2.9	1.8	35.2	0.0	0.1	ZrO_2_ alloyed with Ti
U	0.0	0.0	0.0	0.0	62.5	0.0	1.8	35.4	0.0	0.0	ZrO_2_ substrate

## Data Availability

The original contributions presented in this study are included in the article. Further inquiries can be directed to the corresponding author.
